# Massive Splenomegaly Secondary to Lymphangioma With Gluteal Component in an Adolescent: A Case Report

**DOI:** 10.1155/cris/5705755

**Published:** 2026-10-06

**Authors:** Jonathan F. Guerron-Salazar, Alexis D. Aparicio-Ortiz, Mónica Romero-Macías, Sergio Terriquez-Rodriguez, Margarita Eligio-Angeles

**Affiliations:** ^1^ Department of Pediatric Surgery National Medical Center “20 de Noviembre” Mexico City Mexico; ^2^ Department of Pediatrics National Medical Center “20 de Noviembre” Mexico City Mexico; ^3^ Department of Pathology National Medical Center “20 de Noviembre” Mexico City Mexico

## Abstract

Splenic lymphangioma (SL) is a rare benign lymphatic malformation resulting from abnormal development of lymphatic vessels. It primarily affects children and may coexist with lymphatic malformations at other anatomical sites. Most patients are asymptomatic, and the diagnosis is often incidental. When symptoms occur, they are usually related to the size of the lesion and the compression of adjacent organs. We present the case of a 14‐year‐old male with a history of congenital gluteal and perineal lymphatic malformations, initially misdiagnosed prenatally as myelomeningocele. During follow‐up, the patient developed persistent colicky abdominal pain, progressive abdominal distension, and cutaneous lesions resembling frogspawn, exuding purulent discharge. Physical examination revealed a large, firm, and painful abdominal mass extending from the epigastrium to the hypogastrium. A contrast‐enhanced abdominal computed tomography (CT) scan revealed massive splenomegaly with multiple nonenhancing cystic lesions and displacement of adjacent structures. An open splenectomy was performed due to symptomatic mass effect and diagnostic uncertainty. Intraoperatively, a markedly enlarged, multilobulated spleen was identified and removed without complications. Histopathological examination confirmed the diagnosis of SL, showing multiple thin‐walled cystic spaces filled with eosinophilic proteinaceous material and lined by flat endothelial cells. The patient had an uneventful postoperative course, completed postsplenectomy prophylaxis, and was discharged in stable condition with good oral tolerance, normal bowel and urinary function, and no signs of infection or surgical complications. This case highlights the importance of considering lymphatic malformations in the differential diagnosis of pediatric patients presenting with atypical splenomegaly and cutaneous lymphatic lesions. Early diagnosis and appropriate surgical management can offer both symptomatic relief and definitive diagnostic confirmation in selected cases.

## 1. Introduction

Splenic lymphangioma (SL) is a rare benign lymphatic malformation resulting from abnormal development of the lymphatic channels and is most commonly identified during childhood [1–4]. Although lymphatic malformations occur predominantly in the neck and axillary regions, they may also involve the mediastinum, retroperitoneum, abdominal organs, and, rarely, the spleen [2, 3, 5]. SL is frequently asymptomatic and may be detected incidentally during radiologic examinations [1, 2, 4]. When clinical manifestations occur, they are generally related to the lesion size and compression of adjacent organs [1–4]. Reported manifestations include abdominal pain, nausea, vomiting, loss of appetite, abdominal distension, and a palpable abdominal mass [1, 2, 4]. Large lesions may be associated with complications such as bleeding, consumptive coagulopathy, hypersplenism, portal hypertension, infection, or splenic rupture [1, 2, 6, 7]. The presence of SL in a young patient, particularly when accompanied by cutaneous lymphatic malformations, should prompt assessment for lymphatic involvement at other anatomical sites [2, 3, 7]. Management depends on symptoms, lesion size, distribution, and preservation of functional splenic tissue and may include observation, partial splenectomy, or total splenectomy through open or laparoscopic approaches [1–3, 6]. Herein, we report the case of an adolescent male with congenital gluteal and perineal lymphatic malformations who developed massive symptomatic splenomegaly secondary to SL and was successfully treated with an open total splenectomy.

## 2. Case Presentation

A 14‐year‐old male had an early prenatal ultrasound suggesting a myelomeningocele; however, this diagnosis was excluded at birth, when a lesion compatible with lymphangioma was observed in the right gluteal region.

At 10 months of age, he presented with pain on palpation and movement of the right lower limb. He was evaluated at his local hospital, where the plastic surgery department prescribed analgesics and referred him to a tertiary care center with a presumptive diagnosis of lymphangioma. The lesion resulted in persistent claudication while walking. At the age of five, he underwent Achilles tendon plasty, with poor postoperative recovery despite consistent physical therapy and rehabilitation.

During follow‐up, the patient reported persistent colicky abdominal pain radiating to the right lower limb, which was partially relieved by analgesics. Cutaneous lesions in the perianal and gluteal regions persisted, characterized by heterogeneous, conglomerated, and satellite‐like skin protrusions exuding a malodorous, purulent discharge that resembles frogspawn or frog eggs (Figure 1).

**Figure 1:**
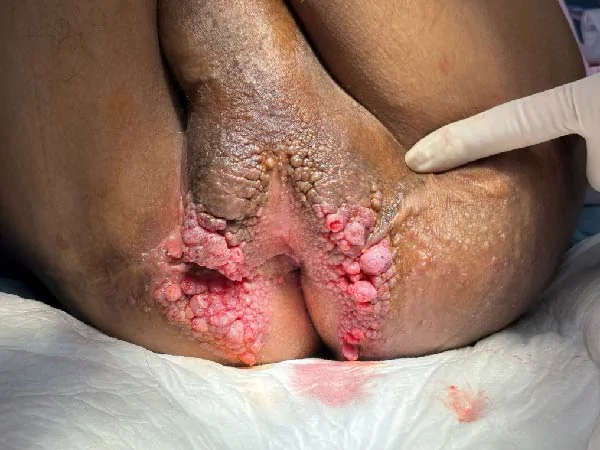
Cutaneous lesion in the intergluteal region characterized by heterogeneous, conglomerated, and satellite‐like skin protrusions exuding malodorous, purulent discharge that resembles frogspawn or frog eggs.

Over time, the lesions worsened, and the patient experienced an acute onset of colicky, belt‐like abdominal pain accompanied by nausea, vomiting, and a fever of 38°C, prompting a referral to our medical center.

On examination, distension of the abdomen was noted due to an abdominal mass extending from 2 cm below the xiphoid process to the pubic symphysis, involving both flanks. The mass was mobile, firm‐to‐stony, painful on palpation, with poorly defined borders, and without peritoneal signs. A contrast‐enhanced abdominal computed tomography (CT) scan revealed marked splenomegaly with displacement of adjacent structures and multiple nonenhancing cystic lesions, some with calcifications (Figure 2A,B).

**Figure 2:**
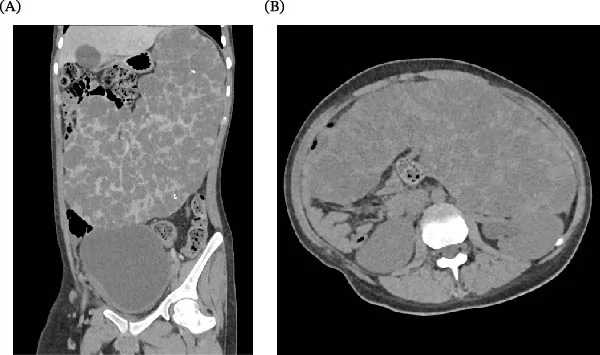
(A) Sagittal contrast‐enhanced abdominal CT image demonstrating a markedly enlarged spleen with lobulated yet well‐defined margins, measuring 30.7 × 22.2 × 16.6 cm and an estimated volume of 5916 cm^3^. The spleen causes displacement of adjacent structures, including the pancreas, left kidney, and hollow viscera. Its parenchyma is replaced by numerous coalescent cystic lesions, some of which exhibit peripheral and septal calcifications. No enhancement is observed following intravenous contrast. (B) Axial contrast‐enhanced abdominal CT image showing the same splenic involvement, with multiple low‐attenuation cystic lesions replacing the splenic parenchyma, without postcontrast enhancement. Adjacent organs are displaced due to the massive splenomegaly.

An open splenectomy was performed via a left subcostal incision under general anesthesia (Figure 3A–C). A markedly enlarged, multilobulated, violaceous spleen extending to the right iliac fossa was mobilized and removed. The splenic vessels, gastrosplenic ligament, and short gastric vessels were ligated and divided, and the specimen was sent for histopathological examination. After irrigation and hemostasis, a drain was placed in the left paracolic gutter, and the wound was closed routinely. The procedure was completed uneventfully, and the patient was transferred to recovery in stable condition.

**Figure 3:**
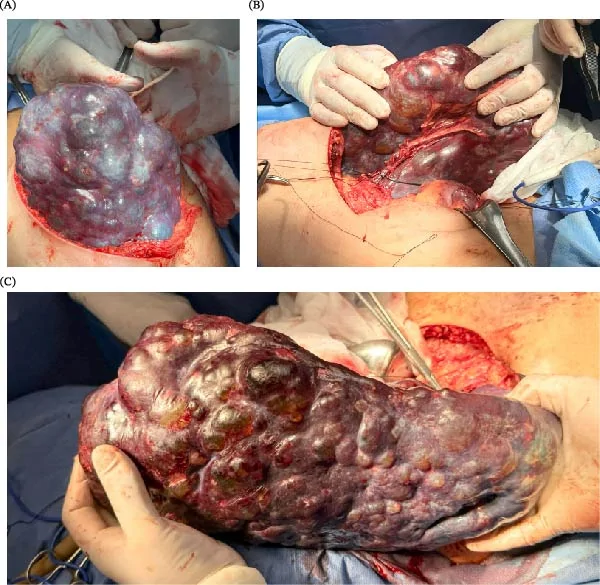
(A) Initial exposure of the markedly enlarged, multilobulated spleen with violaceous discoloration and tense cystic appearance, protruding through the surgical incision. (B) Dissection and ligation of the splenic vessels at the hilum using surgical clamps and sutures. The extensive vascular supply and distorted anatomy due to the massive size of the spleen are evident. (C) Complete removal of the spleen through the surgical incision, demonstrating its multilobulated morphology and translucent cystic lesions, consistent with splenic lymphangioma.

Histopathological analysis revealed a spleen measuring 36 × 25 × 8 cm and weighing 2140 g, with a multinodular external surface and a pale violaceous color. Serial sectioning showed multiple cystic lesions up to 3 cm in diameter filled with serous fluid with smooth and glistening internal walls, consistent with a cystic lymphatic malformation (Figure 4A–D). Immunohistochemical staining for D2‐40 and CD31 was not performed. The diagnosis was established based on the characteristic gross and hematoxylin and eosin (H&E) findings. The postoperative recovery was uneventful. The drain was removed after output decreased, and the patient completed the recommended postsplenectomy antibiotic prophylaxis. At discharge, he was clinically stable, tolerating oral intake, and the surgical wounds were well‐approximated without complications. Follow‐up was scheduled in the outpatient clinic. The main clinical events, diagnostic findings, and therapeutic interventions are summarized in Table 1.

**Figure 4:**
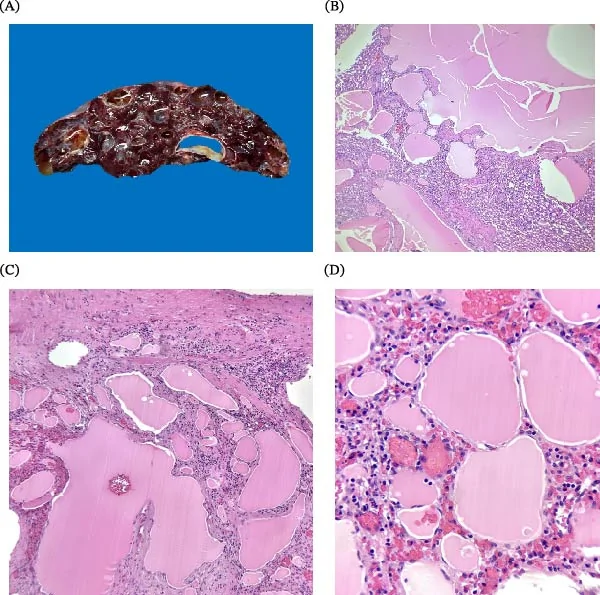
Gross and microscopic pathology of the spleen in a case of splenic lymphangioma. (A) Macroscopic image of the fresh‐cut spleen showing almost complete replacement of the splenic parenchyma by numerous thin‐walled cystic cavities, ranging from 0.2 to 3.0 cm in diameter, filled with clear serous fluid. No areas of necrosis, hemorrhage, or solid lesions are identified. (B and C) Low‐power photomicrographs (H&E, 10×) showing multiple variably sized cystic spaces lined by flat, and in some areas discontinuous or barely discernible, endothelium. The cysts contain eosinophilic proteinaceous material. Residual splenic sinusoids and fibrous stroma are seen between the cystic spaces. (D) High‐power photomicrograph (H&E, 40×) showing dilated lymphatic vessels lined by flat endothelium without cytologic atypia or mitotic activity. Findings are consistent with splenic lymphangioma.

**Table 1:** Clinical timeline of the patient’s presentation, diagnostic evaluation, treatment, and postoperative course.

Time point	Clinical findings, investigations, and interventions
Prenatal period	Prenatal ultrasound suggested myelomeningocele.
Birth	Myelomeningocele was excluded; a right gluteal lesion consistent with a lymphatic malformation was identified.
10 months of age	Pain on palpation and movement of the right lower limb; referral to a tertiary care center with a presumptive diagnosis of lymphangioma.
5 years of age	Achilles tendon plasty followed by rehabilitation, with poor functional recovery.
Subsequent childhood follow‐up	Persistent claudication and progressive gluteal and perineal cutaneous lymphatic lesions.
14 years of age	Acute colicky abdominal pain, nausea, vomiting, fever, abdominal distension, and a large palpable abdominal mass.
Diagnostic evaluation	Contrast‐enhanced CT demonstrated massive splenomegaly with multiple nonenhancing cystic lesions, calcifications, and displacement of adjacent organs.
Surgical treatment	Open total splenectomy was performed because of symptomatic mass effect and marked distortion of the intraabdominal anatomy.
Histopathological evaluation	The spleen measured 36 × 25 × 8 cm and weighed 2140 g. Histological findings were consistent with splenic lymphangioma.
Postoperative period	Uneventful recovery, completion of postsplenectomy prophylaxis, and discharge in stable condition.

Abbreviation: CT, computed tomography.

## 3. Discussion

Massive splenomegaly due to SL is exceptionally rare, particularly in pediatric patients with associated cutaneous lymphatic malformations. Efared et al. [1] reported four cases of SL presenting with massive cystic splenomegaly, with a mean splenic weight of 1675 g and a reported range of 1300–2300 g. In comparison, our patient’s spleen measured 36 × 25 × 8 cm and weighed 2140 g, approaching the upper range reported in that predominantly adult series. This finding is particularly notable given the patient’s pediatric age and explains the abdominal distension, pain, and displacement of adjacent intraabdominal organs observed on imaging.

The diagnosis of SL requires the correlation of clinical, radiologic, and histopathological findings [2–4, 7]. In our patient, the coexistence of congenital gluteal and perineal lymphatic malformations increased the suspicion of a lymphatic lesion. The radiologic differential diagnosis included epithelial and posttraumatic splenic cysts, hydatid disease, abscess, hemangioma, hamartoma, lymphoma, and other vascular splenic neoplasms [2–4]. The presence of multiple nonenhancing cystic lesions replacing most of the splenic parenchyma, some with peripheral and septal calcifications, together with the absence of a solid‐enhancing component, favored a lymphatic malformation [2–4]. Recent radiologic literature describes lymphatic malformations as well‐defined, multilocular cystic lesions that may displace adjacent organs and generally show no internal enhancement, although their walls or septa may enhance [3].

Histologically, SL is characterized by variably sized, thin‐walled lymphatic spaces lined by flattened endothelial cells and containing proteinaceous material, lymphocytes, or occasional erythrocytes [2, 3, 6, 7]. These findings helped distinguish the lesion from hemangioma, in which vascular spaces typically contain red blood cells, and from atypical vascular neoplasms, which may show solid components, cytologic atypia, or increased mitotic activity [2–4]. Immunohistochemical markers, such as D2‐40, CD31, CD34, PROX1, and VEGFR‐3, may support lymphatic endothelial differentiation [3, 6, 7]. However, D2‐40 and CD31 staining was not performed in our patient. Therefore, the diagnosis was established based on the characteristic gross and H&E findings. The absence of these immunohistochemical studies was acknowledged as a limitation rather than being interpreted as evidence against the diagnosis. The recent literature also reports that D2‐40 staining may be partial or absent in some large lymphatic channels [3].

Management depends on symptoms, lesion distribution, and the extent of splenic involvement [1–3, 6]. Observation with radiologic follow‐up may be considered for selected small asymptomatic lesions, whereas partial splenectomy may preserve splenic immune function when the lesion is localized to one pole [3, 6]. Shi et al. reported successful laparoscopic anatomical partial splenectomy for a localized lower‐pole lesion, whereas Pham et al. [2] described successful laparoscopic total splenectomy for symptomatic SL [6]. However, in our patient, the extreme splenic size, extensive replacement of the splenic parenchyma, marked mass effect, and distortion of intraabdominal anatomy favored open total splenectomy. This approach allowed adequate exposure, vascular control, definitive treatment, and complete histopathological evaluation.

The coexistence of splenic and cutaneous lymphatic malformations raises the possibility of multifocal lymphatic involvement. Somatic PIK3CA mutations and activation of the PI3K/AKT pathway have been described in lymphatic malformations and are associated with abnormal tissue growth and persistence of malformed lymphatic lesions [3]. No targeted clinical, genetic, or molecular evaluation for Klippel–Trenaunay syndrome, Maffucci syndrome, or PIK3CA‐related overgrowth spectrum was performed in this patient. Consequently, these conditions cannot be considered definitively excluded. The main strengths of this report are its unusual pediatric presentation, the coexistence of splenic and cutaneous lymphatic malformations, and the radiologic, surgical, and histopathological correlation. Its limitations include the absence of immunohistochemical and molecular confirmation, the lack of targeted assessment for associated syndromic conditions, and limited long‐term follow‐up.

## 4. Conclusion

SL should be considered in pediatric patients with massive cystic splenomegaly, particularly when associated cutaneous lymphatic malformations are present. Correlation of clinical, radiologic, and histopathological findings is essential to distinguish it from other cystic and vascular splenic lesions. In symptomatic patients with extensive splenic involvement and marked distortion of the intraabdominal anatomy, open total splenectomy may provide definitive treatment and diagnostic confirmation. The coexistence of cutaneous and visceral lymphatic malformations should prompt evaluation for additional multifocal involvement.

**Nomenclature**
CT: Computed tomography
H&E: Hematoxylin and eosin
IHC: Immunohistochemistry
SL: Splenic lymphangioma.

## Author Contributions

**Jonathan F. Guerron-Salazar**: case conceptualization, surgical management, data collection, literature review, writing – original draft, intraoperative photography. **Alexis D. Aparicio-Ortiz**: conceptualization, data collection, literature review, writing – original draft, writing – review and editing, preparation of figures, reference management, journal submission and correspondence. **Mónica Romero-Macías**: consultation, review of the clinical section. **Sergio Terriquez-Rodriguez**: surgical management, surgical supervision, consultation. **Margarita Eligio-Angeles**: consultation, histopathological analysis, writing of histological interpretations, analysis of histological slides.

## Funding

No specific funding was received for the research, authorship, or publication of this article.

## Disclosure

This work was conducted as part of the authors’ clinical training and clinical duties at the Department of Pediatric Surgery, the Department of Pediatrics, and the Department of Pathology, National Medical Center “20 de Noviembre,” Mexico City, Mexico.

## Ethics Statement

Institutional ethics committee approval was not required for this single anonymized case report in accordance with institutional policy.

## Consent

Written informed consent for the publication of the patient’s anonymized clinical information and images was obtained from the patient’s parent or legal guardian.

## Conflicts of Interest

The authors declare no conflicts of interest.

## Data Availability

All relevant clinical data supporting the conclusions of this case report are included within the article. No additional datasets were generated or analyzed during the current study.
